# Inhibition of the Mitochondrial Glutamate Carrier SLC25A22 in Astrocytes Leads to Intracellular Glutamate Accumulation

**DOI:** 10.3389/fncel.2017.00149

**Published:** 2017-05-31

**Authors:** Emmanuelle Goubert, Yanina Mircheva, Francesco M. Lasorsa, Christophe Melon, Emanuela Profilo, Julie Sutera, Hélène Becq, Ferdinando Palmieri, Luigi Palmieri, Laurent Aniksztejn, Florence Molinari

**Affiliations:** ^1^INMED, INSERM, Aix-Marseille UniversitéMarseille, France; ^2^Centre De Recherche De L’Institut Universitaire En Santé Mentale de QuébecQuebec City, QC, Canada; ^3^Department of Biosciences, Biotechnologies and Biopharmaceutics, University of Bari, and CNR Institute of Biomembranes, Bioenergetics and Molecular BiotechnologiesBari, Italy; ^4^Aix-Marseille Université, CNRS, IBDM UMR 7288Marseille, France

**Keywords:** mitochondrial glutamate carrier, glutamate transport, epilepsy, astrocytes, glutamate, ATP

## Abstract

The solute carrier family 25 (SLC25) drives the import of a large diversity of metabolites into mitochondria, a key cellular structure involved in many metabolic functions. Mutations of the mitochondrial glutamate carrier *SLC25A22* (also named *GC1*) have been identified in early epileptic encephalopathy (EEE) and migrating partial seizures in infancy (MPSI) but the pathophysiological mechanism of GC1 deficiency is still unknown, hampered by the absence of an *in vivo* model. This carrier is mainly expressed in astrocytes and is the principal gate for glutamate entry into mitochondria. A sufficient supply of energy is essential for the proper function of the brain and mitochondria have a pivotal role in maintaining energy homeostasis. In this work, we wanted to study the consequences of GC1 absence in an *in vitro* model in order to understand if glutamate catabolism and/or mitochondrial function could be affected. First, short hairpin RNA (shRNA) designed to specifically silence *GC1* were validated in rat C6 glioma cells. Silencing *GC1* in C6 resulted in a reduction of the *GC1* mRNA combined with a decrease of the mitochondrial glutamate carrier activity. Then, primary astrocyte cultures were prepared and transfected with shRNA-GC1 or mismatch-RNA (mmRNA) constructs using the Neon® Transfection System in order to target a high number of primary astrocytes, more than 64%. Silencing *GC1* in primary astrocytes resulted in a reduced nicotinamide adenine dinucleotide (Phosphate) (NAD(P)H) formation upon glutamate stimulation. We also observed that the mitochondrial respiratory chain (MRC) was functional after glucose stimulation but not activated by glutamate, resulting in a lower level of cellular adenosine triphosphate (ATP) in silenced astrocytes compared to control cells. Moreover, GC1 inactivation resulted in an intracellular glutamate accumulation. Our results show that mitochondrial glutamate transport via GC1 is important in sustaining glutamate homeostasis in astrocytes.

**Main Points:**

- The mitochondrial respiratory chain is functional in absence of GC1
- Lack of glutamate oxidation results in a lower global ATP level
- Lack of mitochondrial glutamate transport results in intracellular glutamate accumulation

## Introduction

The mitochondrial solute carrier (SLC) family 25 is composed of 53 members that transport a large diversity of metabolites, nucleotides and cofactors across the inner mitochondrial membrane (IMM; Palmieri, 2004, 2013; Palmieri and Monné, 2016). These transporters are essential for mitochondria where several metabolic pathways occur including the Krebs cycle and the β-oxidation of fatty acids. More importantly, mitochondria are essential for cellular energy homeostasis synthesizing adenosine triphosphate (ATP) by oxidative phosphorylation. Fourteen diseases have been associated with a dysfunction of a specific member of the SLC25 family (Palmieri, 2013, 2014) and four of them are involved in the mitochondrial glutamate transport.

Mutations in the *SLC25A12*, which encodes the mitochondrial aspartate-glutamate carrier isoform 1 (AGC1), were identified in patients presenting a developmental delay, hypotonia and intractable seizures associated with a global hypomyelination in the brain (Wibom et al., 2009; Falk et al., 2014). The activity of the mutant proteins AGC1 was severely reduced by ~85% or completely abolished (Wibom et al., 2009; Falk et al., 2014). The study of the *Agc1*-knockout mice showed a similar phenotype with a developmental delay, pronounced motor deficits with an impaired myelination in the central nervous system resulting in a premature death at 3 weeks (Jalil et al., 2005; Sakurai et al., 2010). The second AGC isoform (AGC2, SLC25A13) is associated with neonatal and adult-onset type II citrullinemia, an autosomal recessive disease caused by a liver-specific loss of argininosuccinate synthetase activity (for review, see Chanprasert and Scaglia, 2015). The loss of AGC2 could be compensated by AGC1 in many organs but not in the liver where it is the only isoform expressed (del Arco et al., 2002). The Slc25a13-null mice showed a reduced mitochondrial aspartate transport in the liver but no apparent *in vivo* phenotype (Sinasac et al., 2004). This absence of phenotype was due to a high mitochondrial glycerol-3-phosphate dehydrogenase (Gpd2) activity in the mouse liver, an enzyme that is much less active in human (Williams et al., 1986; Sadava et al., 2004). The *Slc25a13/Gdp2* double-knockout developed citrullinemia, hyperammonemia, hypoglycemia and fatty liver and is a more representative mouse model of the human disease (Saheki et al., 2007). A complete loss of activity of the mitochondrial glutamate carrier 1 (GC1, SLC25A22) has been associated with early epileptic encephalopathy (EEE; Molinari et al., 2005, 2009; Cohen et al., 2014) and migrating partial seizures in infancy (MPSI; Poduri et al., 2013), but, to the best of our knowledge, no animal model has been developed for this human pathology.

The glutamate carrier isoforms GC1 and GC2 (SLC25A18) are symporters that catalyze the transport of glutamate associated with a proton (H^+^) through the IMM (Fiermonte et al., 2002). Once in the internal space of the mitochondria, glutamate is converted by glutamate dehydrogenase (GDH) into α-ketoglutarate and ammonia together with the reduction of nicotinamide adenine dinucleotide (Phosphate) (NAD(P)^+^) into NAD(P)H that enters into complex I of the respiratory chain. The mRNA expression level of GC1 is higher than that of GC2 in many tissues, notably liver, pancreas and kidney; however, their mRNA levels are similar in the brain (Fiermonte et al., 2002). Furthermore, the Km and Vmax values of GC1 are higher than those of GC2 (5.2 vs. 0.26 mM; 12.2 vs. 3.9 μmol/min/g of proteins, respectively, Fiermonte et al., 2002). In the light of their mRNA level expressions and kinetic parameters, it seems that, when expressed in the same cell, GC2 is responsible for the basic function of glutamate degradation and that GC1 becomes operative to accommodate higher demands. Glutamate can also enter into mitochondria using AGC1 and 2 which combine the input of glutamate to the release of aspartate (Palmieri et al., 2001). As GC1 is highly expressed in pancreas, Casimir et al. (2009) silenced GC1 in insulinoma INS-1E cells and demonstrated the important physiological function of this carrier in the control of glucose-stimulated insulin secretion. However, although mutations of GC1 leading to a complete loss of function have been reported in patients with EEE or MPSI (Molinari et al., 2005, 2009; Poduri et al., 2013), no study of GC1 inhibition in cerebral cells has been performed hitherto. Several protein expression analyses in the rodent brain showed that AGC1 and AGC2 are almost completely restricted to neurons (Ramos et al., 2003; Berkich et al., 2007; Xu et al., 2007), while GC1 is highly expressed in astroglial cells from different structures (retina, spinal cord, cortex; Berkich et al., 2007). Therefore, GC1 represents the principal gate for glutamate entry into the mitochondria of astrocytes.

In this work, the main goal was to study if the absence of GC1 could affect mitochondria functions and particularly ATP synthesis. For this purpose, an *in vitro* model, primary astrocyte cultures from rat cortical cortices, was used to study the biochemical consequences of GC1 inhibition with a particular interest in glutamate and mitochondrial metabolism. Our results show that the GC1 knock-down induced by short hairpin RNA (shRNA) abolishes NAD(P)H production upon glutamate stimulation. We also observed that the mitochondrial respiratory chain (MRC) is fully activated by glucose but not by glutamate resulting in a decrease of the cytosolic ATP level. Finally, we showed that GC1 inactivation results in intracellular glutamate accumulation.

## Materials and Methods

### shRNA Constructs

Three small interfering RNA (siRNA) were designed from the rat *GC1* cDNA sequence (NM_001014027) in the coding sequence (shRNA-GC1.C: nucleotides 707–727) or in the 3′-untranslated region (3′UTR: shRNA-GC1.D and E: nucleotides 958–977 and 1846–1865, respectively). BLAST searches confirmed the target specificity of these constructs. As negative control, we used a scrambled mismatch RNA (mmRNA, GATGAACCTGATGACGTTC, gift from Dr. C. Pellegrino). These sh/mmRNA were subcloned into a mU6Pro vector (gift from Dr. J. Lo Turco). For the stable clone, we inserted a puromycin cassette into this vector (gift from Dr. C. Beclin).

### Cell Culture

Rat C6 glioma cells were cultured at 37°C under a humidified atmosphere with 5% CO_2_ with a complete medium DMEM supplemented with 10% FBS (Sigma) and 100 units/mL antibiotics/antimycotics.

Primary cultures of rat astrocytes were obtained from cortices of the embryonic day 18 (E18) Sprague-Dawley rats, according to protocols approved by the Comité National de Réflexion Ethique sur l’Expérimentation Animale (n°14) N°92-20122012. After the removal of the hippocampi, cortices were first enzymatically disrupted with trypsin 2.5% for 15 min at 37°C and then mechanistically disrupted by pipetting several times. After centrifugation at 400 *g* for 5 min, cells were resuspended in DMEM:F12 (1:1) supplemented with 20% FBS, 1 mM sodium pyruvate, 100 units/mL antibiotics/antimycotics. This suspension was filtered with a 70 μm cell stainer and cells were seeded in poly-ethyleneimine coated flasks and maintained in DMEM-20% FBS at 37°C (5% CO_2_) for 1 week. Then, rate of FBS was reduced to 10% and cells were maintained in culture during 2–3 more weeks and then used for experiments. All culture products come from Life Technologies/Thermo Fisher Scientific Incorporation.

### Transfection

Cells were transfected using the Neon® Transfection System (Life Technologies) according to the manufacturer’s protocol. Briefly, cells were trypsinized and counted with the cell counter SCEPTER (Millipore). For astrocytes, 100,000 cells in suspension were transfected with a total amount of 1 μg of DNA containing a reporter plasmid encoding mRFP1 alone or in combination with sh or mmRNA constructs (ratio 1:5), with the following configuration: 1400 V, 1 pulse, 30 ms. Then, cells were cultured on pre-coated glass coverslips and maintained at 37°C and 5% CO_2_ with a medium supplemented with 10% FBS for 2 days before imaging experiments.

For C6 cells and quantitative PCR, 10^6^ cells were electroporated with a total amount of 10 μg DNA at 1860 V, 1 pulse, 20 ms and cultured on 25 cm^2^ flasks during 2 days before RNA extraction.

### Immunocytochemistry

Astrocytes grown on coverslips were rinsed with phosphate buffered saline (PBS) and fixed with cold antigenfix solution (Diapath) for 15 min at room temperature (RT). Cells were then rinsed in PBS and permeabilized with 0.3% Triton-PBS supplemented with 10% goat serum for 1 h at RT. Primary GFAP antibody (monoclonal mouse anti-rat, 1:500, Millipore) was incubated overnight at 4°C in 0.3% Triton-PBS supplemented with 3% goat serum. Cells were rinsed and incubated with Alexa 488-conjugated goat anti-mouse (monoclonal, 1:500, Life Technologies) 2 h at RT in the same solution. Coverslips were mounted in Vectashield with DAPI (Vector Laboratories, Burlingame, CA, USA) and observed with an ApoTome microscope (Zeiss).

### RT-PCR and Quantitative PCR

Total RNA was isolated from C6 using RNeasy Plus Mini kit and cDNA was synthesized using the Quantitect Reverse Transcription kit, according to the manufacturer’s protocol (QIAGEN). Quantitative PCR (qPCR) was performed on a Light cycler 480 using SYBR-Green chemistry (Roche) and specific primers for *Rattus norvegicus (Rn) GC1* (QIAGEN, QT00420420) and *Rn Rpl13a* (QT00425873) as control. qPCR were performed with 5 μL of diluted cDNA template, specific primers (0.6 μM) and SYBR Green I Master Mix (7.5 μL) at a final volume of 15 μL. Each reaction was performed at an annealing temperature of 60°C and for 50 cycles. Reactions were performed in duplicate and melting-curve analysis was performed to assess the specificity of each amplification. A standard curve was performed for each gene with a control cDNA diluted at different concentrations. Relative expression was assessed with the calculated concentration in respect to the standard.

### Mitochondrial Isolation

C6 cells were lysed by Dounce glass homogenization on ice in a hypotonic buffer containing 3.5 mM Tris/HCl, 2.5 mM NaCl and 0.5 mM MgCl_2_, pH 7.4. Lysed cells were then suspended in an isotonic buffer (35 mM Tris/HCl, 25 mM NaCl and 5 mM MgCl_2_, pH 7.4) and mitochondrial fraction was isolated by serial centrifugations, as previously described (Wieckowski et al., 2009). Mitochondria were then washed with ice-cold STE buffer (250 mM sucrose, 20 mM Tris/HCl, 1 mM EDTA, pH 7.4) and then subjected to immunoblotting or transport activity assays.

### Electrophoresis and Western Blot

Cellular or mitochondrial proteins were solubilized in a buffer containing 2% SDS, 0.15 M sucrose, 0.1 M Tris/HCl, pH 7.4 and subjected to a 15% SDS polyacrylamide gel electrophoresis. Proteins were then transferred onto nitrocellulose membrane and incubated for 2 h at RT or at 4°C overnight with either an anti-GC1 (1:3000) antiserum, the anti-AGC1 (monoclonal mouse antibody, 1:5000, BD Biosciences, Franklin Lakes, NJ, USA), the anti-β-ATPase antibody (monoclonal mouse antibody, 1:1000, BD Biosciences), the anti-firefly luciferase (polyclonal rabbit antibody, 1:1000, Thermo Fisher Scientific) and anti-GAPDH (monoclonal mouse antibody, 1:500, Merck Millipore), respectively. Anti-GC1 antiserum was generated in rabbit against the peptide CDVVKTRLQSERGVN corresponding to amino acids 246–261 of rat GC1 sequence as synthetic antigen (Eurogentec, Seraing, Belgium). Protein levels were revealed by chemiluminescent HRP Immobilon Western kit (Millipore Corp., Billerica, MA, USA) after incubation for 2 h at RT with an anti-rabbit IgG antibody (1:10,000 for GC1, 1:3000 for Luciferase) or anti-mouse IgG antibody (1:1000 for AGC1 and β-ATPase, 1:3000 for GAPDH) conjugated to horseradish peroxidase (Thermo Scientific, Rockford, IL, USA).

### Glutamate Transport Activity in Liposomes

Transport activity in liposomes was assayed as previously described (Palmieri et al., 1995) with minor modifications. Briefly, isolated mitochondria from control C6 cells and stable clones were solubilized (0.5 mg/ml) in a buffer containing 3% (w/v) Triton X-114, 1 mM EDTA, and 10 mM PIPES at pH 6.5 for 30 min at 0°C and centrifuged at 138,000 *g* for 30 min. The preparation used for reconstitution consisted of 100 μL of solubilized mitochondria (~40 μg of protein), 70 μl of 10% (w/v) Triton X-114, 90 μl of 10% (w/v) egg yolk phospholipids (Fluka) in the form of sonicated liposomes, 20 mM L-glutamate, 10 mM MES (pH 6.0), 0.6 mg of cardiolipin (Sigma), and water to a final volume of 700 μL. After vortexing, this preparation was recycled 13 times through the hydrophobic column Sm-2 Biobeads (BioRad) column equilibrated with 10 mM MES (pH 6.0) and 20 mM L-glutamate. After the external substrate was removed by Sephadex chromatography, transport at 25°C was started by the addition of 1 mM of L-[^14^C(U)]glutamate (Perkin Elmer, NEC 290E with a specific activity of 260 mCi/mmol) or L-[^14^C(U)]aspartate (Perkin Elmer, NEC 268E with a specific activity of 260 mCi/mmol) and terminated by addition of 15 mM pyridoxal 5′-phosphate and 10 mM bathophenanthroline according to the inhibitor-stop method (Fiermonte et al., 2009). The incubation time was 15 min. A volume of 10 μL of 11 mM labeled glutamate or 0.55 mM labeled aspartate solution (with a specific activity of about 5400 cpm/nmol and 110,000 cpm/nmol, respectively) was added to samples of 100 μl of eluted proteoliposomes. In controls, the inhibitors were added at the beginning together with the external labeled substrate. Finally, the external substrate was removed, and the radioactivity inside the liposomes was measured. The experimental values were corrected by subtracting control values (Palmieri et al., 1995).

### NAD(P)H Measurement

NAD(P)H generation was assessed in astrocytes transfected with mRFP1 alone (control), shRNA-GC1.C + mRFP1 or mmRNA + mRFP1. The mRFP1 construct was used to identify transfected cells in order to perform single cell analysis. Astrocytes seeded on coverslips (~100,000 cells) were maintained during 10 min in a resting condition, i.e., a low glucose KRBH (Krebs-Ringer bicarbonate-HEPES buffer) medium containing (in mM): 135 NaCl, 3.6 KCl, 10 Hepes, 5 NaHCO_3_, 0.5 NaH_2_PO4, 0.5 MgCl_2_, 1.5 CaCl_2_ and 0.5 Glucose (Glc) ± 200 μM DL-threo-ß- Benzyloxyaspartic acid (DL-TBOA; gift from the NIMH Chemical Synthesis and Drug Supply Program). Then, a 200 μM-glutamate stimulation was performed. NAD(P)H assays were performed on an inverted microscope Nikon TE 300, recorded with Metamorph software (7.1.7.0, Molecular Devices) and NAD(P)H autofluorescence was measured using excitation and emission filters set at 350 and 490 nm, respectively. Images were taken during resting condition and after glutamate stimulation. The experimental values were corrected by subtracting the background of each image. The NAD(P)H autofluorescence was normalized after the 10 min stimulation period in KRBH-0.5mM Glc, without glutamate, by setting the fluorescence at 100%.

### Mitochondrial Membrane Potential Measurement (∆ψm)

Mitochondrial membrane potential (∆ψm) was assessed with Rhodamine 123 (R123, Life Technologies), a fluorescent probe that enters into active mitochondria. First, astrocytes were washed 10 min with KRBH-0.5 mM glucose, incubated with R123 (5 μg/mL) for 30 min at RT and washed again three times in KRBH-0.5 mM glucose. Coverslips seeded with ~100,000 astrocytes were placed on an inverted microscope Nikon DIAPHOT and the transfected cells (red) correctly loaded with R123 were selected. Acquisition was performed with a Hamamatsu Orca-ER camera and R123 fluorescence (λexc = 488 nm; λem = 520 nm) was followed with the Simple PCI software (1 image each 30 s). Astrocytes were perfused with the KRBH-0.5 mM glucose, resting condition, to set the fluorescence at 100% and then stimulated with glutamate (1 mM), glucose (15 mM) and carbonylcyanide p-trifluoromethoxyphenylhydrazone (FCCP; 1 μM, from Sigma-Aldrich), consecutively. The experimental values were corrected by subtracting the background of each image. Each cell was analyzed individually and all the results were pooled together at the end. The integrated area under each normalized curve was calculated after each stimulation using the OriginPro software.

### Cytosolic ATP Measurement

Cytosolic ATP levels were monitored in astrocytes expressing the ATP-sensitive bioluminescent probe luciferase. Astrocytes were transfected with the Luciferase-pcDNA3 alone (control), in the presence of shRNA or mmRNA and seeded on a 24-wells plate (~300,000 cells per wells). Experiments were performed 2 days after transfection. The Luciferase-pcDNA3 was a gift from William Kaelin (Addgene plasmid # 18964). First, astrocytes were washed 15 min with KRBH-0.5 mM glucose at RT, incubated with 100 μM beetle luciferin (Promega) for 30 min at RT and then the 24-well plate was placed into a thermostated plate reader (FLUOstar OPTIMA, BMG Labtech) in the luminometer mode. After a 10 min period incubation in basal KRBH-0.5 mM glucose (resting condition) to set the fluorescence at 100%, cells were stimulated with glutamate (1 mM), 30 min later with glucose (15 mM) and finally FCCP (10 μM) was added. The experimental values, corresponding to a population of cells, were corrected by subtracting the background of each acquisition. The integrated area under each normalized curve was calculated after each stimulation using the OriginPro software.

### HPLC Analysis for Amino Acid Determination

Amino acid determination was assessed in astrocytes transfected with mRFP1 alone (control), shRNA-GC1.C/D + mRFP1 or mmRNA + mRFP1. Transfected astrocytes were seeded on 4-wells plate (~300,000 cells per wells) and experiments were performed 48 h after transfection. First, astrocytes were maintained in KRBH-0.5 mM glucose (30 min, resting condition), then stimulated with glutamate (1 mM) for 30 min or 1 h, and finally glucose (15 mM) was added. After each condition (resting condition, glutamate or glucose stimulations), culture medium was removed and cells were washed once with cold PBS and collected by scrapping in 150 μL of lysis buffer (in mM: 10 Hepes, 200 NaCl, 2.5 MgCl_2_, 2 CaCl_2_, 5 EDTA, 1.5% Triton, Protease/Phosphatase Inhibitors, pH 7.4). Thirty microliters of each were taken for protein concentration using BCA method. The remaining 120 μL were immediately frozen and stored at −80°C until assayed. Amino acids determination was performed as previously described (Re et al., 2006). Briefly, the day of assay, 30 μL of samples were incubated with 30 μL of perchloric acid (0.5 M) for 10 min at 4°C and then centrifuged at 15,000 *g* for 10 min to precipitate proteins. Supernatants were collected and amino acids levels were determined using a Waters high-performance liquid chromatography (HPLC) fluorometric detection system (Waters, Milford, MA, USA). Data were computed with the Millenium software from Waters, identification and quantification of peaks were achieved by comparison with standard solutions. Concentrations of each amino acid were divided by the total protein concentration.

### Statistics

Data are represented as means ± SEM and the number of experiments is indicated. When the data’s distribution was normal, we used a Student’s *t*-test to compare means of two groups, paired or unpaired, or the one-way ANOVA followed by Bonferroni or Tukey-Kramer’s test as mentioned. When the normality test failed, we used the non-parametric Wilcoxon signed test for paired samples or Mann-Whitney test for two related or unrelated groups, respectively, or the Kruskal-Wallis followed by Dunn test for multiple comparisons. ns: not significant; *p < 0.05; **p < 0.01; ***p < 0.001.

## Results

### GC1 Silencing in Rat C6 Glioma Cells

**Figure 1:**
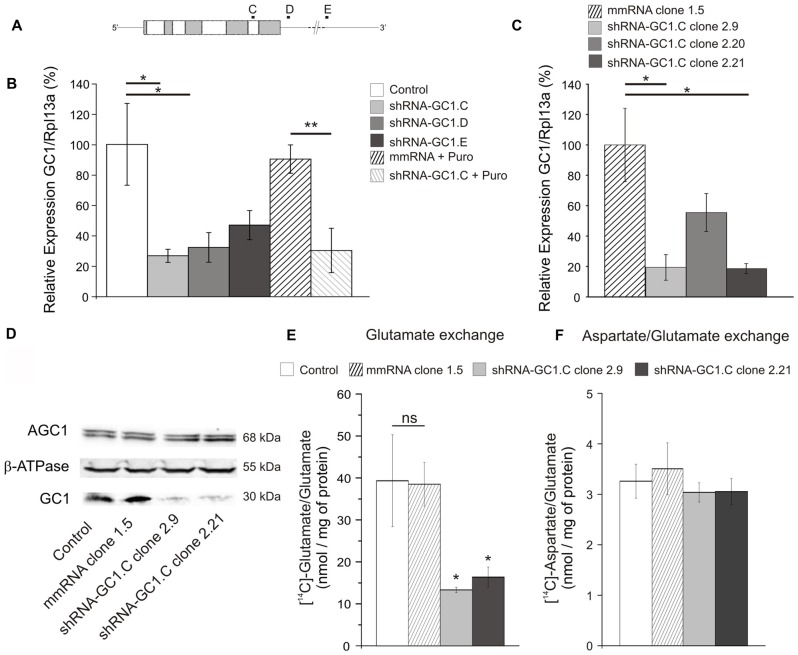
***Glutamate carrier (GC1)* expression analyses and glutamate transport activity after short hairpin RNA (shRNA) transfection in C6 cell line. (A)** Graphical representation of *GC1* mRNA from rat (2534 pb) and localization of the three different shRNA-GC1.C, D and E Gray and white boxes represent the nine exons of *GC1* gene. **(B,C)** Relative expression level of *GC1*, normalized with the expression level of the ribosomal protein L13A (Rpl13A), in C6 cells transfected transiently **(B)** or in stable C6 clones **(C)**. **(B)** Compared with control cells (transfected with mRFP1 alone), *GC1* expression is decreased by 73.4, 67.8 and 53.1% with constructs C, D and E, respectively (*n* = 6). After the insertion of the puromycin resistance gene into the vector shRNA-GC1.C, *GC1* expression is still decreased by 69.8%, whereas the mismatch RNA (mmRNA) is not different from the control cells (*n* = 3). **(C)** In stable C6 clones, compared with mmRNA-clone 1.5, *GC1* expression is decreased by 80.6, 44.5 and 81.4% with clones shRNA-GC1.C-2.9, 2.20 and 2.21, respectively (*n* = 4). Data are means ± SEM. Unpaired *t*-test vs. Control or mmRNA. **(D)** Protein expression level analyses of stable clones 2.9 and 2.21 by Western blot. Twenty-five micrograms of mitochondrial proteins from C6 wild-type (Control) and stable clones (mismatch 1.5, shRNA-GC1.C-2.9 and 2.21) were separated on 15% SDS-PAGE and then transferred onto nitrocellulose membrane for immunodetection with the anti-GC1 antiserum, anti-aspartate-glutamate carrier isoform 1 (AGC1) monoclonal antibody and anti-β-ATPase antibody, respectively. Densitometry analysis revealed that GC1 content was drastically reduced, about 80%, in 2.9 and 2.21 clones in comparison with WT and mismatch clone, while similar levels of AGC1 protein were detected in each tested sample in three separated experiments. **(E,F)** Glutamate and aspartate transport activities were assayed in liposomes reconstituted with about 40 μg of mitochondrial proteins from control C6 and stable clones (mismatch, 2.9 and 2.21 clones) and containing 20 mM glutamate (*n* = 3). Transports were started by adding 1 mM [^14^C]glutamate **(E)** or 0.05 mM [^14^C]aspartate **(F)** to reconstituted liposomes containing 20 mM glutamate. After 15 min of incubation, the uptake of labeled substrates was stopped by addition of 15 mM pyridoxal 5′-phosphate and 10 mM bathophenanthroline. Glutamate transport is significantly decreased in the two clones stably inhibited for GC1 (by 66 and 58%, respectively), whereas aspartate/glutamate exchange is equivalent in each cell type. Data are expressed as means ± SEM. One-way ANOVA followed by Bonferroni’s test.

With the aim of studying the effect of GC1 knockdown in astrocytes, three shRNA, targeting the coding sequence or the 3′-UTR (Figure 1A), and a mmRNA, an ineffective shRNA as a control, were generated. Each construct was transiently transfected into a rat C6 glioma cell line to evaluate their efficiency to inactivate endogeneous *GC1* expression. Using quantitative PCR, we found that *GC1* expression was significantly reduced by shRNA-GC1.C and D (respectively: reduction of 73.4%, *p* = 0.02 and 67.8%, *p* = 0.04, Figure 1B) while in mmRNA transfected cells, *GC1* mRNA expression level was not different from the control cells. To confirm these results at the protein level, a high concentration of GC1-inactivated mitochondria was needed to perform Western blotting. For this purpose, a puromycin cassette was inserted into shRNA-GC1.C and mmRNA vectors, without affecting their inhibition efficiency (Figure 1B), and several C6 stable clones with a very low GC1 expression were generated (clones 2.9 and 2.21, Figure 1C), as confirmed by Western blot analysis (Figure 1D).

Then, the activity of the mitochondrial glutamate carrier GC1 in mitochondrial extracts from these C6 clones was tested by measuring the rate of [^14^C]glutamate/glutamate exchange in reconstituted liposome (Fiermonte et al., 2002). We observed that the uptake of 1 mM radioactive glutamate was significantly reduced in liposomes reconstituted with shGC1-stable clones (13.3 ± 0.6 and 16.4 ± 2.4 nmol/mg of protein for clones 2.9 and 2.21, respectively) compared to liposomes reconstituted with control cells or mmRNA-stable clone (39.3 ± 6.3 and 38.5 ± 5.2 nmol/mg of protein respectively, Figure 1E). Because in C6 glioma the glutamate/glutamate exchange could also be catalyzed by the aspartate/glutamate carriers (AGC1 and 2), the aspartate/glutamate exchange, performed only by AGC, was measured in the same experimental conditions. We observed that the aspartate/glutamate exchange in liposomes reconstituted with shGC1.C-stable clones was similar to the control cells in contrast to the glutamate/glutamate exchange (Figure 1F). This result shows that, under our experimental conditions, absence of glutamate transport by GC1 is compensated neither by GC2 nor by AGC1-2, carriers which are typically very poorly expressed in astrocytes (Berkich et al., 2007).

### Silencing GC1 in Astrocytes Reduces NAD(P)H Production

**Figure 2:**
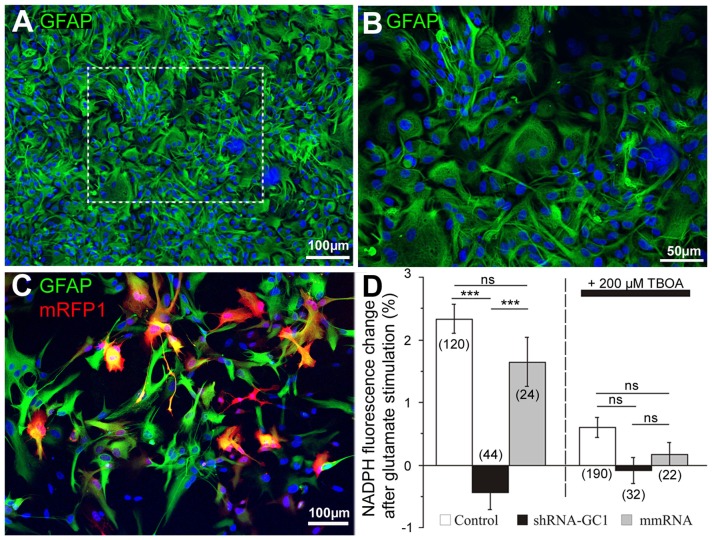
**Efficient shRNA-GC1 transfection of primary cortical astrocyte cultures resulted in reduced nicotinamide adenine dinucleotide (Phosphate) (NAD(P)H) production. (A,B)** Immunolocalization of glial fibrillary astrocytes protein (GFAP; green) on a primary culture of cortical astrocytes from E18 rat in **(A)** and at a higher magnification **(B)**. Nuclei are labeled with DAPI (blue). **(C)** A primary astrocyte culture observed 48 h after mRFP1 transfection with the Neon Transfection System (1400 V, 1 pulse, 30 ms). Astrocytes are labeled with GFAP (green) and transfected cells are in red. **(D)** NAD(P)H autofluorescence was monitored in astrocytes transfected with mRFP1 (control), shRNA-GC1 or mmRNA. Fluorescence level was measured in resting condition (0.5 mM glucose) to set the fluorescence to 100% and measured 10 min after glutamate stimulation (200 μM). Left graph: after glutamate stimulation, NAD(P)H fluorescence levels in control and mmRNA cells were increased (2.3 ± 0.2% and 1.6 ± 0.4% respectively) but almost no modification in GC1-silenced astrocytes (−0.4 ± 0.3%). Right graph: similar experiment but in the presence of 200 μM DL-threo-ß-Benzyloxyaspartic acid (DL-TBOA). When glutamate could not enter into astrocytes, NAD(P)H fluorescence is stable in each group of astrocytes. The number of cells is indicated below each group. Experiments were performed with five independent transfections derived from three individual astrocyte culture batches. Data are expressed as mean ± SEM. Kruskal-Wallis followed by Dunn test.

In order to study the inhibition of GC1 specifically in astrocytes, primary astrocyte cultures were prepared and the purity of these cultures was checked by immunocytochemistry using the glial fibrillary acidic protein (GFAP) antibody (Figure 2). In our culture, 86.1% GFAP positive cells (±1.2, *n* = 583 cells, Figures 2A,B) were found. Then, astrocytes were transfected with mRFP1 alone or combined with shRNA-GC1.C or mmRNA using a powerful transfection method, the Neon® Transfection System (Life Technologies). This system allowed us to transfect 64.1% astrocytes (±2.1%, *n* = 1019 cells, Figure 2C) using the optimal protocol of one pulse at 1400 V for 30 ms. This system was a valuable tool for the experiments in astrocyte cultures reported below.

GC1 catalyzes the transport of glutamate into the mitochondria where it is mainly catabolized by the action of GDH. This reaction results in the formation of NAD(P)H, α-ketoglutarate and ammonia. Thus, in the absence of GC1, we expected that application of glutamate would not be associated with an increase of NAD(P)H in mitochondria. To measure NAD(P)H production after glutamate stimulation in primary astrocyte cultures, we used its natural optical properties. Indeed, the reduced form of this molecule, NAD(P)H, absorbs light (λexc = 320–380 nm) and emits fluorescence (λem = 420–480 nm) whereas the oxidized form, NAD(P)^+^, does not. All the experiments in this study were performed in a medium containing a concentration of glucose (0.5 mM) close to the physiological conditions in rat brain [0.47 mM (Fellows et al., 1992)]. Since the concentration of glutamate rises dramatically during neurotransmission (from ~25 nM to ~1 mM), we chose glutamate concentration between this range to stimulate glutamate transporters (Danbolt, 2001; Herman and Jahr, 2007). Application of stimulatory 200 μM glutamate increased the NAD(P)H levels by 2.3% in control astrocytes (±0.2%) and by 1.6% in astrocytes transfected with the mmRNA (±0.4%, Figure 2D). These values are similar to those observed by Casimir et al. (2009) in INS-1E cells with glucose stimulation. To ensure that this increase was really linked to glutamate import into astrocytes, 200 μM DL-TBOA, a specific membrane glutamate transporter (excitatory amino acid transporter, EAAT) inhibitor (Shimamoto et al., 1998) that blocks glutamate entry into astrocytes, was added. Under this condition, no significant increase of NAD(P)H fluorescence was observed (Figure 2D). Furthermore, the same experiments were performed in astrocytes transfected with shRNA-GC1 where we did not observe any increase of NAD(P)H, in the presence or the absence of DL-TBOA (Figure 2D). Therefore, reduction of GC1 expression resulted in a decrease of mitochondrial glutamate transport activity with no neo-NAD(P)H formation. Transfection of mmRNA had no consequence on NAD(P)H formation, further supporting that this observation is specific to GC1 inactivation.

### Mitochondrial Respiratory Chain Is Functional but Not Activated by Glutamate in Knocked-Down GC1 Astrocytes

**Figure 3:**
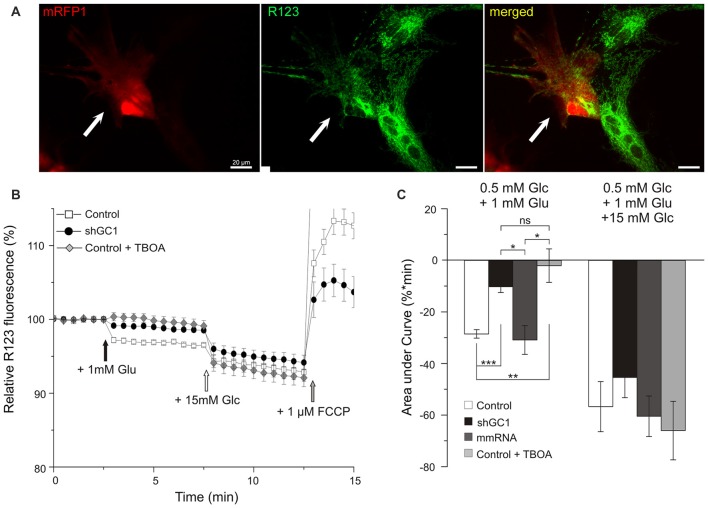
**Mitochondrial respiratory chain (MRC) is functional but not activated by glutamate in astrocytes knocked-down for GC1. (A)** Relative mitochondrial membrane potential (∆ψm) was assessed in transfected astrocytes (red, left, white arrow) loaded with Rhodamine 123 (R123, green, middle) in a medium containing low glucose (0.5 mM, resting condition). **(B)** Mitochondrial membrane hyperpolarization was elicited by addition of 1 mM glutamate (Glu, black arrow) and by 15.5 mM Glucose (Glc, white arrow). Control depolarization was assessed with the uncoupler Carbonyl cyanide-4-(trifluoromethoxy)phenylhydrazone (FCCP; 1 μM, gray arrow). Measurements were made in control astrocytes (white squares, *n* = 28), transfected with shRNA-GC1 (black circles, *n* = 23) or in the presence of DL-TBOA (gray diamonds, *n* = 29). Data are normalized with the resting condition and expressed as means ± SEM at each time point. **(C)** Area under normalized curves was measured after each stimulation in control astrocytes in the presence or not of DL-TBOA, in astrocytes transfected with shRNA-GC1 or mmRNA (*n* = 12). Data are expressed as mean ± SEM. Kruskal-Wallis followed by Dunn test. Experiments were performed with 4–10 independent transfections, derived from 10 individual astrocyte cultures.

NADH is the substrate of complex I of the MRC and its oxidation results in the translocation of four protons (H^+^) across the membrane, thus producing a proton gradient. To determine if the MRC was functional in the absence of GC1, the ∆ψm was analyzed. ∆ψm reflects the pumping of H^+^ across the inner membrane during the process of electron transport and oxidative phosphorylation. For this purpose, the fluorescent probe Rhodamine123 (R123), a cell-permeant dye that is sequestered by active mitochondria, was loaded into astrocytes (Figure 3A). At high concentrations, this positively charged probe accumulates within the mitochondrial matrix, which is negatively charged, and forms aggregates, thus quenching some of the fluorescence emission. Hyperpolarization of the ∆ψm will result in a higher sequestration of R123 and to a relative decrease of the fluorescence level (Kahlert et al., 2008; Perry et al., 2011). Conversely, a sustained depolarization will result in a release of the dye, increasing the R123 signal. The protocol applied consisted of a first stimulation with 1 mM of glutamate alone to observe if the MRC could be activated by this substrate, and then a second stimulation with 15 mM of glucose to verify that the MRC was operative. Indeed, we have previously shown that, in the absence of GC1, the MRC was functional and could be activated by other substrates (Molinari et al., 2005).

In control astrocytes, the glutamate stimulation resulted in a decrease of fluorescence that reached the level of 96.7% (±0.09%, Figure 3B) corresponding to a hyperpolarization of the ∆ψm. Supplementation of 15 mM glucose resulted in a greater hyperpolarization with a plateau at 93.3% (±0.5%). These values are in accordance with those observed in previous studies using INS-1E β cells (Merglen et al., 2004; Casimir et al., 2009). Finally, a further addition of the protonophore FCCP (1 μM) resulted in a rapid depolarization, reflecting the proton gradient dissipation (Figure 3B). In GC1 inactivated astrocytes (Figure 3B), glutamate stimulation resulted in a much lower hyperpolarization since the fluorescence reached the plateau of 98.7% (±0.1%, Figure 3B) while 15 mM glucose stimulation resulted in a greater hyperpolarization (94.6 ± 0.4%). In the presence of DL-TBOA (Figure 3B), the hyperpolarization was only induced by 15 mM glucose (92.1% ± 0.5) and not by glutamate addition. Areas under the curve (AUC) were calculated after each stimulation and confirmed a much lower hyperpolarization after glutamate stimulation in inactivated GC1 cells with a decrease of 64 and 67% compared to control and mismatch cells, respectively (Figure 3C). In presence of TBOA, AUC after glutamate stimulation was not significantly different from the inactivated GC1 cells (Figure 3C). Moreover, we observed that the glucose addition resulted in a similar hyperpolarization in each condition compared to the control cells (Figure 3C). These observations indicate that, in the absence of GC1, the MRC is fully functional but not activated by the addition of glutamate alone.

### The Global ATP Level Is Lower in Knocked-Down GC1 Astrocytes after Glutamate Stimulation

**Figure 4:**
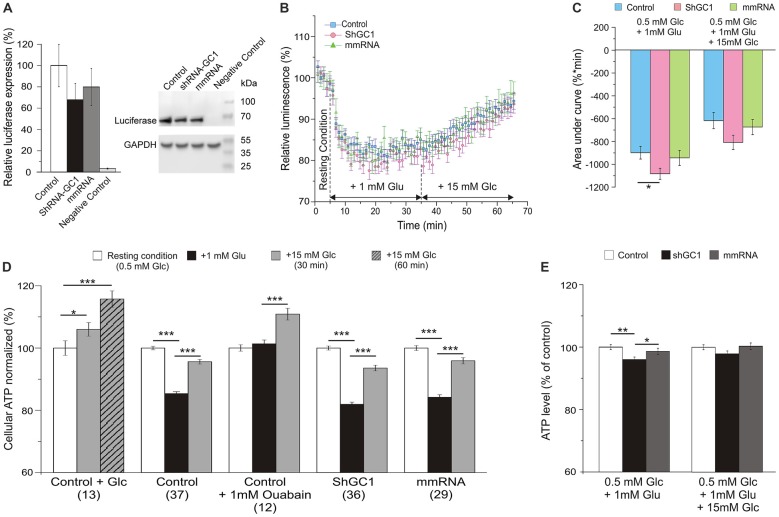
**Cellular adenosine triphosphate (ATP) level is lower in astrocytes inactivated for GC1 after glutamate stimulation**. Astrocytes were transfected with luciferase alone (Control), co-transfected with shRNA-GC1 or with mmRNA, and loaded with beetle luciferin (100 μM) in a low Glucose-containing medium to monitor cellular ATP level. **(A)** Relative luciferase expression level, normalized with GAPDH expression level, in Control, GC1 inactivated and mmRNA cells (*n* = 3). Luciferase expression was equivalent in the three conditions and completely absent from non-transfected cells (Negative Control). **(B)** The luminescence intensity was measured in Control astrocytes (blue squares, *n* = 37), transfected with shRNA-GC1 (pink circles, *n* = 36) or mmRNA (green triangles, *n* = 29) during resting condition (0.5 mM glucose), after glutamate (Glu, 1 mM) and then glucose (Glc, 15 mM) stimulations. Data were normalized with the resting condition. **(C)** Area under normalized curves was measured after each stimulation in control astrocytes, transfected with shRNA-GC1 or mmRNA. Data are expressed as mean ± SEM. One-way ANOVA followed by Tukey-Kramer test.** (D,E)** Summary of the luminescence intensity measurements, normalized with the resting condition **(D)** or relative to control cells of each condition **(E)**. Data are means of the three last minutes in each condition ± SEM. The number of wells is indicated below each condition. Kruskal-Wallis followed by Dunn test **(D)** and One-way ANOVA followed by Tukey-Kramer test **(E)**. Experiments were performed with 3–12 independent transfections and derived from four individual astrocyte cultures.

Since the proton gradient across the IMM is used to enhance ATP synthesis in the process of oxidative phosphorylation through the ATP synthase, we wondered if this synthesis was disturbed in our model. In this study, intracellular ATP levels were measured by monitoring the light produced by the luciferase-luciferin reaction. First, the expression level of luciferase was evaluated by Western blot and a similar expression was observed in cells transfected with luciferase alone or with shRNA-GC1 or mmRNA (Figure 4A). As a positive control, ATP synthesis was assessed in the presence of glucose only. Addition of 15 mM glucose in control astrocytes resulted in an increase of global ATP level 30 min later with a level of luminescence of 105.3 (±1.4%) and a greater increase 1 h after (116.6 ± 1.8%, Figure 4D). Similar experiments were then performed but with glutamate stimulation (1 mM) and a sustained decrease of global ATP was observed 30 min later in control (85.4 ± 0.7%), GC1 inactivated (81.9 ± 0.7%) and mismatch cells (84.2 ± 0.8%, Figures 4B,D). The expression of the data as percentage of corresponding controls showed that this decrease of ATP was greater in absence of GC1 (96.0 ± 0.8%, *p* = 0.0026, Mann-Whitney test, Figure 4E). In contrast, the addition of 15 mM glucose, 30 min after glutamate, resulted in an equivalent increase of ATP in the three different conditions (level of luminescence in control: 95.6 ± 0.7%; shGC1: 93.6 ± 0.9%; mmRNA: 95.9 ± 1.0%; Figures 4B,E). AUC were calculated after each stimulation and confirmed a lower ATP level in inactivated astrocytes compared to control cells (Figure 4C). This difference was no longer significant after glucose stimulation (Figures 4C,E). As a control, addition of FCCP resulted in a rapid decrease of cytosolic ATP levels (55.7, 55.2 and 55% of decrease in control, shGC1 and mmRNA respectively after 5 min, data not shown).

As shown in several studies, the entry of glutamate is associated with three molecules of Na^+^ resulting in the activation of the Na^+^/K^+^-ATPase and to a consumption of ATP (Barbour et al., 1991; Grewer and Rauen, 2005; Magistretti and Chatton, 2005). In order to demonstrate that the ATP decrease, observed after glutamate stimulation, was due to the activation of the Na^+^/K^+^-ATPase, a similar experiment was performed in control cells but in the presence of 1 mM ouabain, in order to inhibit all the Na^+^/K^+^-ATPase isoforms. Under these conditions, no ATP consumption was observed after glutamate stimulation (Figure 4D). These observations show that, in the absence of GC1, the balance between ATP consumption and synthesis is perturbed only in the presence of glutamate. After glucose stimulation, this equilibrium is similar in each condition.

### Glutamate Level Is Increased in Knocked-Down GC1 Astrocytes

**Figure 5:**
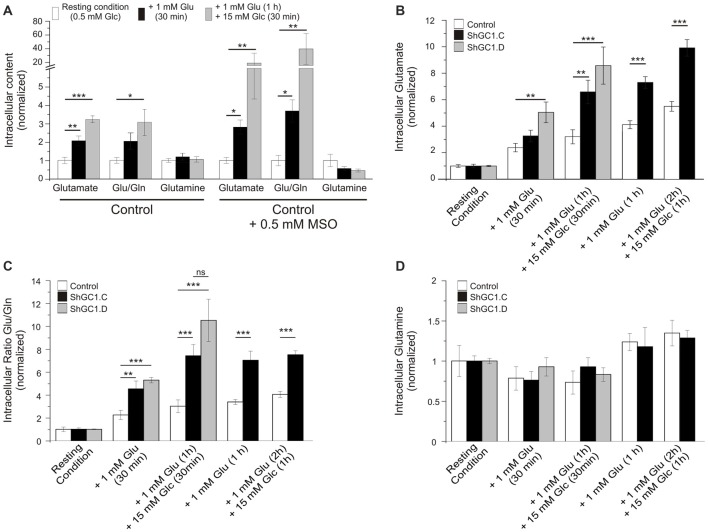
**Glutamate level is increased in GC1-deficient astrocytes. (A)** Control astrocytes were incubated in KRBH-low glucose (0.5 mM, resting condition) or in the presence of L-Methionine sulfoximine (MSO, 0.5 mM) in order to inhibit the glutamine synthetase (GS). Astrocytes were stimulated with glutamate (Glu, 1 mM) for 30 min and then glucose (Glc, 15 mM) were added for 30 min more. The cellular content of glutamate and glutamine (Gln) was determined by high-performance liquid chromatography (HPLC) and the ratio Glu/Gln was calculated. **(B–D)** Similar experiments but in absence of MSO. Control and GC1 knock-down astrocytes were incubated in resting condition for 30 min, supplemented with 1 mM Glu for 30 min or 1 h, and then 15 mM Glc were added for 30 min or one more hour. The cellular content of Glu and Glutamine (Gln) was analyzed in control, transfected astrocytes with shRNA-GC1.C or D **(B,D)** and the ratio Glu/Gln was calculated **(B)**. Each experimental condition consists of 6–10 samples from 10 independent transfections derived from five individual cell culture batches. Data are expressed as means ± SEM. Kruskal-Wallis followed by Dunn test **(A)** and unpaired *t*-test vs. Control in each condition **(B–D)**.

Once in the astrocytes, glutamate could be metabolized by multiple enzymes including glutamine synthetase (GS), alanine amino transferase (ALAT), aspartate amino transferase (AAT) and mitochondrial GDH (Westergaard et al., 1996; Sonnewald et al., 1997; McKenna et al., 2000). In the absence of glutamate entry into mitochondria, we wondered if glutamate would accumulate in astrocytes and if the level of glutamine would be affected. In preliminary experiments, we measured the glutamate and glutamine levels in control cells, in absence or presence of the GS inhibitor L-Methionine sulfoximine (L-MSO, 0.5 mM), in order to determine if we could detect any change in the glutamate level with HPLC. Indeed, in the absence of GS activity, glutamate accumulation in astrocytes has been demonstrated in both hippocampal astrocytes and Müller cells of the retina with immunogold labeling (Laake et al., 1995; Barnett et al., 2000; Perez et al., 2012). In absence of L-MSO, the application of glutamate increased the glutamate/glutamine ratio by 2.1 (±0.2) after 30 min and by 3.3 (±0.2) after 1 h (Figure 5A). This increase is due to an increase in the glutamate level without modification of the glutamine level. In MSO treated cells, an increase by 2.8 (±0.4) in the glutamate/glutamine ratio was also observed after 30 min of glutamate application but this effect was more pronounced after a 1 h glutamate application with a glutamate/glutamine ratio of 39.3 ± 22.7 linked to a glutamate level increase combined with a decrease of glutamine level (Figure 5A). As glutamate accumulation could be determined by HPLC, we then performed glutamate and glucose stimulations on astrocytes transfected with mRFP1 alone (control) or with shRNA-GC1.C (Figures 5B–D) but in absence of L-MSO. In GC1 inactivated cells, a progressive increase of the intracellular glutamate concentration was observed (Figure 5B), following glutamate stimulation alone (1 mM, 30 min or 1 h) or glutamate and then glucose addition (15 mM, 30 min or 1 h). The glutamate/glutamine ratio increased significantly in astrocytes inactivated for GC1 compared to control cells, without any significant change in glutamine level (Figures 5C,D). Similarly to glutamine, the alanine level was unchanged, whatever the stimulation (Table 1); by contrast, aspartate tended to increase with glutamate stimulation duration without significant differences between GC1 knocked-down and control astrocytes (Table 1). In order to confirm that these results were due to GC1 inactivation, we performed same experiments but with the shRNA-GC1.D. We observed the same consequences, i.e., an increase of the ratio glutamate/glutamine linked to an increase of the glutamate level but without any significant changes in the other amino acids (Figures 5B–D and Table 1). Therefore, the absence of GC1 has a direct consequence on intracellular glutamate level, without affecting the other amino acids, at least those tested here.

**Table 1:** **Aspartate and alanine contents determined by high-performance liquid chromatography (HPLC) in astrocytes transfected with mRFP1 alone (Control, *n* = 8), with shRNA-GC1.C (*n* = 8) or with shRNA-GC1.D (*n* = 10)**.

		Control	ShRNA-GC1.C	ShRNA-GC1.D
		Mean SEM	Mean SEM	Mean SEM
Aspartate	Resting condition	1.000 ± 0.166	1.000 ± 0.152	1.000 ± 0.140
	+ Glutamate (30 min)	0.645 ± 0.280	0.630 ± 0.225	0.741 ± 0.054
	+ Glutamate (1 h)	1.178 ± 0.516	1.449 ± 0.614	1.723 ± 0.065
	+ Glutamate (1 h) + Glucose (30 min)	1.309 ± 0.148	1.266 ± 0.098	
	+ Glutamate (2 h) + Glucose (1 h)	1.173 ± 0.134	1.250 ± 0.090	
Alanine	Resting Condition	1.000 ± 0.162	1.000 ± 0.214	1.000 ± 0.222
	+ Glutamate (30 min)	0.674 ± 0.231	0.455 ± 0.137	0.725 ± 0.128
	+ Glutamate (1 h)	0.572 ± 0.220	0.415 ± 0.124	0.525 ± 0.103
	+ Glutamate (1 h) + Glucose (30 min)	0.968 ± 0.175	0.828 ± 0.118	
	+ Glutamate (2 h) + Glucose (1 h)	0.695 ± 0.023	0.652 ± 0.065	

*Data are normalized on the resting condition and expressed as mean ± SEM*.

## Discussion

Glutamate homeostasis is crucial for the proper functioning of the central nervous system and its extracellular concentration is maintained low thanks to the astroglial glutamate transporters GLT-1 and GLAST (EAAT 1 and 2; Takahashi et al., 1997; Danbolt, 2001; Robinson and Jackson, 2016). As described in several studies, EAAT activity and cycling rate are dependent on intracellular ionic composition (in particular on Na^+^) and high intracellular glutamate concentrations reduced extracellular glutamate uptake (Barbour et al., 1991; Otis and Jahr, 1998; Bergles et al., 2002; Grewer and Rauen, 2005; Wadiche et al., 2006; Tzingounis and Wadiche, 2007). Therefore, the intracellular glutamate concentration into astrocytes is finely regulated by multiple enzymes. Most of the glutamate is metabolized by GS, in the cytosol, or by GDH, in mitochondria (Westergaard et al., 1996; Sonnewald et al., 1997; McKenna et al., 2000; Danbolt, 2001). The balance between the extent of oxidative consumption of glutamate (reflecting mitochondrial glutamate catabolism) and synthesis of glutamine by GS is dependent on extracellular glutamate concentration, with relatively more glutamate being oxidized at higher glutamate concentrations (from 15% to 43% as extracellular glutamate concentration increased from 0.1 mM to 0.5 mM in primary culture of cortical astrocytes; McKenna et al., 2000). In rat cerebellar slices, about 70% of extracellular glutamate is converted by GS (~40%) and by the GDH (~30%; de Barry et al., 1983). The entry of glutamate into mitochondria is achieved by a mitochondrial glutamate carrier subfamily which comprises two members, GC1 and GC2 (SLC25A22 and SLC25A18) that are equally expressed in the brain (Fiermonte et al., 2002; Palmieri, 2004). Whereas GC1 protein expression has been shown in astrocytes, the presence of GC2 could not be assessed (Berkich et al., 2007). In this study, we showed that inhibition of GC1 results in the absence of NAD(P)H formation and in the non-activation of the MRC when glutamate is the only substrate. This result confirms that GC1 is drastically reduced in our transfected cells and shows that this carrier is the main gate for net glutamate entry into mitochondria of primary astrocyte cultures. So far, the absence of glutamate entry into astrocytes’ mitochondria results in reduced ATP concentration and, finally, to an intracellular accumulation of glutamate.

The decrease of ATP level observed in GC1 inactivated cells compared to control cells might be caused by the non-activation of the MRC or, alternatively, by an increase of ATP consumption via the activation of enzymatic processes in order to consume the excess of intracellular glutamate. In the cytosol of astrocytes, two energetic-dependent processes could be requested: (i) the glutamine synthesis via the GS, a cytosolic enzyme expressed only in astrocytes which converts glutamate and ammonia into glutamine via ATP consumption (Sonnewald et al., 1997; Schousboe and Waagepetersen, 2003); and (ii) the glutathione (GSH) synthesis, a two-step reaction, catalyzed by the gamma-cysteine synthase (γ-GCS) and the GSH synthase in an ATP-dependent manner (DeLeve and Kaplowitz, 1991). In our experimental conditions, the intracellular glutamine levels were unchanged, suggesting that excess of glutamine is exported in the extracellular space or that GS is already fully active. Our results correlate with those obtained by Skytt et al. (2012) in an *in vitro* model inactivated for GDH where glutamate level greatly increased with no modification of glutamine level. Dringen and Hamprecht (1996) showed that the addition of 1 mM glutamate to astrocytes increased GSH concentration only if cysteine and glycine were also added. The absence of these substrates in our medium suggests that this pathway is not stimulated. The transport of extracellular glutamate into the intracellular space of astrocytes, performed by EAAT, is a highly consuming energy process as it is sensitive to Na^+^ and K^+^ electrochemical gradients, and the activation of the Na^+^/K^+^-ATPase is essential to maintain these gradients (Pellerin and Magistretti, 1994; Magistretti and Pellerin, 1999; Chatton et al., 2000; Magistretti and Chatton, 2005). Recent studies showed that mitochondria were observed in close vicinity to glutamate transporters and formed a macromolecular complex with the Na^+^/K^+^-ATPase, leading to the concept that mitochondria are recruited to fuel active transport of glutamate with direct glutamate oxidation (Rose et al., 2009; Genda et al., 2011; Bauer et al., 2012; Jackson et al., 2014, 2015; Ugbode et al., 2014). Moreover, pharmacological inhibition of GDH resulted in an inhibition of the glutamate and gamma-aminobutyric acid (GABA) uptake in crude cortical membranes, suggesting that mitochondrial GDH provides energy for transport using endogenous glutamate (Whitelaw and Robinson, 2013). The finding that the global concentration of ATP after glutamate stimulation is lower in astrocytes inactivated for GC1 compared to control cells suggests that processes requiring ATP could be altered in the absence of GC1. The conversion of glutamate into glutamine by GS is an ATP-dependent reaction (Sonnewald et al., 1997; Schousboe and Waagepetersen, 2003) and its absence or inhibition results in a rapid decrease of the glutamine level (Laake et al., 1995; He et al., 2010; Trabelsi et al., 2017). The observation that the glutamine level was maintained in the absence of GC1 shows that GS is functional and suggests that ATP processes are still operational, at least under our conditions.

In view of our findings, we hypothesize that inactivation of enzymes/proteins involved in astrocytic glutamate metabolism can have similar consequences to those observed in the absence of GC1 and may result in similar pathologies, i.e., EEEs or MPSI (Molinari et al., 2005, 2009; Poduri et al., 2013; Cohen et al., 2014). Interestingly, GS deficiency caused glutamate accumulation *in vivo* (Laake et al., 1995; Perez et al., 2012) and was associated with mesial temporal lobe epilepsy (Eid et al., 2004) and EEE (Häberle et al., 2005, 2011). A recent study showed that glutamate conversion into glutamine via GS is important to limit the extracellular glutamate spill-over and the activation of the peri/extrasynaptic NMDA receptors (Trabelsi et al., 2017). The authors recorded astrocytes from juvenile rat neocortical slices and showed that, after GS inhibition with L-MSO, synaptically transporter current (STC) evoked by high frequency stimulation was twice slower than STC evoked from saline injected rats, and that NMDAR-excitatory postsynaptic currents were larger with a strong peri/extrasynaptic component in pyramidal cells (Trabelsi et al., 2017). These results suggest that a rapid clearance of extracellular glutamate is compromised when glutamate catabolism in astrocytes is altered. Moreover, we have previously shown that a defect in glutamate clearance leads to the generation of epileptic-like discharges *in vitro* and a burst-suppression pattern in rat pups (Cattani et al., 2007; Milh et al., 2007; Molinari et al., 2012). Therefore, glutamate accumulation in astrocytes might be the starting point for network hyper-excitability. In order to study the consequences of the absence of GC1 within a neuronal network, GC1 should be inactivated *in vivo* or *ex vivo* in brain slices. However, transfection of astrocytes is of limited effectiveness in intact tissue, and drugs known to inactivate GC1 are not specific (Fiermonte et al., 2002) making the analysis hazardous and inaccurate. In future studies, because of the lack of an *in vivo* model, we would like to develop a recombinant adeno-associated viral vector containing a shRNA-GC1 in order to study the electrophysiological consequences of GC1 absence in the neuronal network.

## Author Contributions

FM: conception of the research, statistical analysis; EG, YM, FML, CM, EP, JS, HB and FM: data acquisition; EG, YM, FML, FP and FM: data analysis and interpretation; FP, LP, FML, LA and FM: drafting of the work and critical revising.

## Conflict of Interest Statement

The authors declare that the research was conducted in the absence of any commercial or financial relationships that could be construed as a potential conflict of interest.

## Abbreviations

AAT: aspartate amino transferase
AGC: aspartate/glutamate carriers
ALAT: alanine amino transferase
ATP: aenosine triphosphate
∆ψm: mitochondrial membrane potential
DL-TBOA: DL-threo-ß-Benzyloxyaspartic acid
EAAT: excitatory amino acid transporter
EEE: early epileptic encephalopathy
FCCP: Carbonyl cyanide-4-(trifluoromethoxy)phenylhydrazone
GABA: gamma-aminobutyric acid
GC1: glutamate carrier 1
GDH: glutamate dehydrogenase
GFAP: glial fibrillary acidic protein
Glc: glucose
Gln: glutamine
Glu: glutamate
GS: glutamine synthetase
GSH: glutathione
HPLC: high-performance liquid chromatography
IMM: inner mitochondrial membrane
L-MSO: L-Methionine sulfoximine
mmRNA: mismatch-RNA
MPSI: migrating partial seizures in infancy
MRC: mitochondrial respiratory chain
NAD(P)+/NAD(P)H: nicotinamide adenine dinucleotide (Phosphate)
R123: Rhodamine123
shRNA: short hairpin RNA
SLC25: solute carrier family 25
STC: synaptic transporter current.
